# Severe Hypocalcemia and Hypophosphatemia Following Ferric Carboxymaltose in a Patient With Roux-en-Y Gastric Bypass

**DOI:** 10.7759/cureus.115655

**Published:** 2026-09-02

**Authors:** Ahmed Elhassan, Isra Ali

**Affiliations:** 1 Endocrinology, Mohammed Bin Rashid University of Medicine and Health Sciences, Dubai, ARE; 2 Biomedical Sciences, Mohammed Bin Rashid University of Medicine and Health Sciences, Dubai, ARE

**Keywords:** ferric carboxymaltose, fgf23, hypocalcemia, roux-en-y gastric bypass surgery, severe hypophosphatemia

## Abstract

Iron deficiency anemia is commonly treated with intravenous iron when oral therapy is ineffective, poorly tolerated, or inadequately absorbed. Ferric carboxymaltose (FCM) is an effective intravenous iron formulation but is increasingly recognized to cause hypophosphatemia through fibroblast growth factor 23 (FGF23)-mediated renal phosphate wasting. Although FCM-induced hypophosphatemia is well described, concurrent clinically significant hypocalcemia is less commonly reported. We present the case of a 39-year-old woman with a history of Roux-en-Y gastric bypass, iron deficiency anemia, and vitamin D deficiency who developed severe symptomatic hypocalcemia and hypophosphatemia following a course of FCM. She presented with generalized muscle cramps, paresthesia, perioral numbness, blurry vision, and fatigue and required repeated hospitalization and intensive electrolyte replacement. Laboratory evaluation demonstrated severe hypocalcemia and hypophosphatemia, elevated C-terminal FGF23, secondary hyperparathyroidism, and inappropriate urinary phosphate loss. Her previous bariatric surgery and vitamin D deficiency likely increased her susceptibility to FCM-associated disturbances in mineral metabolism. This case highlights the importance of identifying patients at increased risk before FCM administration and considering biochemical monitoring after treatment, particularly in individuals with altered gastrointestinal anatomy or pre-existing nutritional deficiencies.

## Introduction

Iron deficiency anemia is a common complication following bariatric surgery, particularly Roux-en-Y gastric bypass, because of reduced dietary intake and impaired absorption of iron and other micronutrients. Oral iron replacement may therefore be ineffective or poorly tolerated in these patients, making intravenous iron therapy an important treatment option.

Ferric carboxymaltose (FCM) is an intravenous iron formulation that permits the administration of relatively large doses of iron over a short period and is generally well tolerated. However, hypophosphatemia is an increasingly recognized adverse effect of FCM. The principal mechanism involves an increase in biologically active fibroblast growth factor 23 (FGF23). Elevated FGF23 decreases proximal renal tubular phosphate reabsorption, resulting in increased urinary phosphate excretion. It also suppresses renal synthesis of 1,25-dihydroxyvitamin D, thereby reducing intestinal absorption of phosphate and calcium [1,2].

The severity of FCM-associated hypophosphatemia varies considerably. Although many cases are mild and transient, severe or prolonged hypophosphatemia may cause fatigue, muscle weakness, bone pain, and, in cases of persistent phosphate depletion, osteomalacia and fractures [2,3]. Certain populations appear to be at increased risk, including patients with vitamin D deficiency, hyperparathyroidism, malabsorption, and previous bariatric surgery [4-6].

Patients who have undergone Roux-en-Y gastric bypass may be particularly susceptible because the procedure predisposes them to chronic disturbances in calcium and vitamin D absorption and secondary hyperparathyroidism [6]. These underlying abnormalities may reduce their ability to compensate for changes in phosphate and vitamin D metabolism induced by FCM. Studies involving patients with previous bariatric surgery have demonstrated an increased risk of FCM-associated hypophosphatemia, accompanied by increased urinary phosphate excretion and changes in FGF23 concentrations [4,5].

Although hypophosphatemia following FCM administration is increasingly recognized, the simultaneous occurrence of profound symptomatic hypocalcemia is less commonly reported. We describe a patient with previous Roux-en-Y gastric bypass and vitamin D deficiency who developed severe hypocalcemia and hypophosphatemia following FCM administration, requiring repeated hospitalization and intensive electrolyte replacement.

## Case presentation

A 39-year-old woman with a history of obesity had undergone Roux-en-Y gastric bypass approximately six years before presentation. Her subsequent medical history was notable for iron deficiency anemia and vitamin D deficiency. Following bariatric surgery, she had initially taken regular vitamin and calcium supplementation but had discontinued regular supplementation several years before presentation. Because of iron deficiency anemia requiring parenteral replacement and intolerance of oral iron, she received FCM (Ferinject) 500 mg weekly for five weeks, corresponding to a cumulative dose of approximately 2,500 mg.

Following FCM therapy, she presented to the emergency department with generalized muscle cramps, paresthesia, blurry vision, perioral numbness, fatigue, and difficulty walking. Initial laboratory investigations demonstrated profound hypocalcemia and hypophosphatemia, with a serum calcium level of 5.3 mg/dL (reference range: 8.9-10.2 mg/dL) and serum phosphate level of 0.8 mg/dL (reference range: 2.7-4.5 mg/dL). Serum albumin was 3.7 g/dL (reference range: 3.4-4.8 g/dL), parathyroid hormone (PTH) was 70.0 pg/mL (reference range: 6.2-29.0 pg/mL), 25-hydroxyvitamin D was 19.4 ng/mL, and magnesium was 1.69 mg/dL (reference range: 1.7-2.55 mg/dL). The biochemical findings and subsequent course are summarized in Table 1.

**Table 1:** Biochemical findings at presentation and during subsequent evaluation *The laboratory defines 25-OH vitamin D concentrations of 30-100 ng/mL as sufficient. PTH: parathyroid hormone; FGF23: fibroblast growth factor 23; 25-OH vitamin D: 25-hydroxyvitamin D

Parameter	Initial presentation	Subsequent course/evaluation	Reference range	Interpretation
Serum calcium	5.3 mg/dL	Repeatedly low during early follow-up (approximately 5.5-8.3 mg/dL), with subsequent normalization documented	8.9-10.2 mg/dL	Severe hypocalcemia with persistent/recurrent abnormalities
Serum phosphate	0.8 mg/dL	Repeatedly low during follow-up (approximately 1.1-2.6 mg/dL), with later normalization documented	2.7-4.5 mg/dL	Severe and recurrent hypophosphatemia
Albumin	3.7 g/dL	-	3.4-4.8 g/dL	Normal
PTH	70.0 pg/mL	-	6.2-29.0 pg/mL	Elevated
25-OH vitamin D	19.4 ng/mL	Later 32.3 ng/mL	30-100 ng/mL*	Initially low, subsequently sufficient
Magnesium	1.69 mg/dL	Subsequently improved	1.7-2.55 mg/dL	Mildly decreased initially
1,25-dihydroxyvitamin D	-	54.4 pg/mL	19.9-79.3 pg/mL	Normal when measured
C-terminal FGF23	-	156 RU/mL	26-110 RU/mL	Elevated
24-hour urinary phosphate	-	1,316.7 mg/24 h	400-1,300 mg/24 h	Inappropriately high in the setting of hypophosphatemia
24-hour urinary calcium	-	72.6 mg/24 h	100-300 mg/24 h	Decreased

An electrocardiogram (ECG) obtained during the presentation demonstrated sinus rhythm with a corrected QT interval (QTc) of 451 ms (Figure 1).

**Figure 1:**
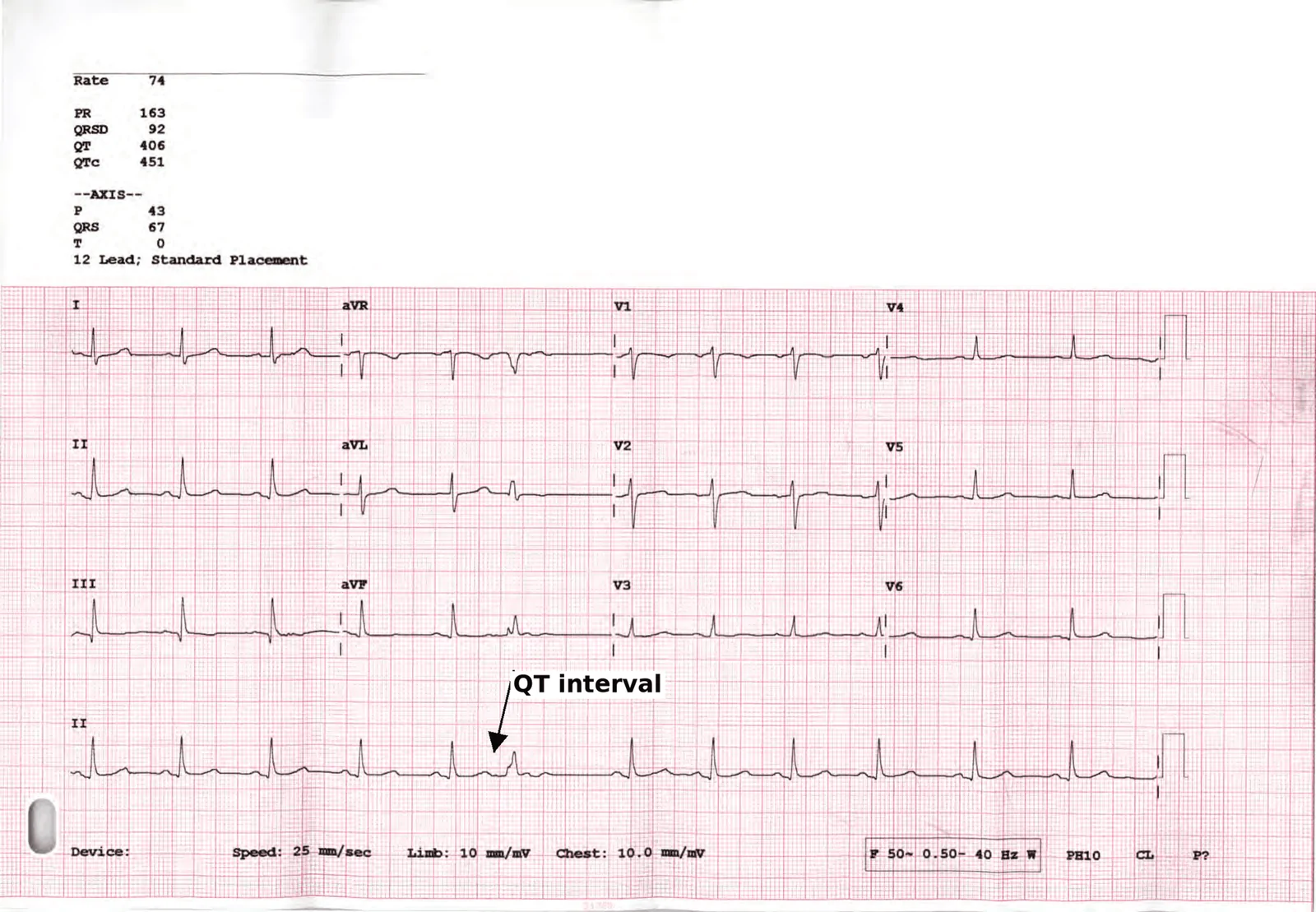
Electrocardiogram obtained during the patient's presentation with hypocalcemia

Subsequent evaluation demonstrated persistent hypophosphatemia with inappropriate urinary phosphate loss. Twenty-four-hour urinary phosphate excretion was 1,316.7 mg/24 h despite ongoing hypophosphatemia, and C-terminal FGF23 was elevated at 156 RU/mL. Twenty-four-hour urinary calcium was reduced at 72.6 mg/24 h, while 1,25-dihydroxyvitamin D was within the laboratory reference range at 54.4 pg/mL. These findings, in the context of recent FCM exposure, were consistent with an FGF23-associated renal phosphate-wasting mechanism.

The patient was initially treated with intravenous calcium and phosphate replacement with concurrent magnesium repletion and was subsequently transitioned to oral supplementation following symptomatic and biochemical improvement. Her symptoms subsequently recurred, requiring further hospital treatment and intravenous electrolyte replacement. Serial measurements demonstrated persistent and recurrent hypocalcemia and hypophosphatemia during the subsequent clinical course.

Longer-term management included calcium, phosphate, vitamin D3, alfacalcidol, and magnesium supplementation. With continued replacement and monitoring, her symptoms improved and the biochemical abnormalities gradually resolved.

## Discussion

This case demonstrates severe concurrent hypophosphatemia and hypocalcemia following FCM administration in a patient with previous Roux-en-Y gastric bypass and vitamin D deficiency. The temporal relationship with FCM exposure, together with profound hypophosphatemia, inappropriate urinary phosphate loss, and elevated C-terminal FGF23, was consistent with an FGF23-associated renal phosphate-wasting mechanism. The profound hypocalcemia was likely multifactorial, reflecting the interaction between FCM-associated disturbances in mineral metabolism and pre-existing susceptibility related to vitamin D deficiency, impaired mineral absorption following Roux-en-Y gastric bypass, secondary hyperparathyroidism, and mild hypomagnesemia.

FCM-associated hypophosphatemia is primarily mediated by FGF23. In a randomized trial comparing FCM with ferumoxytol, FCM markedly increased biologically active FGF23, resulting in renal phosphate wasting, decreased calcitriol and calcium concentrations, and increased PTH [2]. FGF23 acts on the proximal renal tubule to decrease the expression of sodium-phosphate cotransporters, thereby increasing urinary phosphate loss. It also suppresses renal 1alpha-hydroxylase activity, reducing the synthesis of 1,25-dihydroxyvitamin D. Reduced active vitamin D further decreases intestinal phosphate absorption and may impair calcium absorption. The resulting decrease in serum calcium can stimulate PTH secretion, which may further contribute to renal phosphate wasting [1,2]. In our patient, however, 1,25-dihydroxyvitamin D was within the laboratory reference range when measured later during the evaluation; therefore, biochemical suppression of this metabolite was not demonstrated at that time.

The effect of FCM on FGF23 and phosphate metabolism has also been demonstrated in randomized comparisons with other intravenous iron preparations. Wolf et al. found a substantially higher incidence of hypophosphatemia following FCM than following iron isomaltoside, now known as ferric derisomaltose [1]. Although many episodes are asymptomatic and transient, severe or prolonged hypophosphatemia can occur, particularly following repeated exposure or in patients with additional risk factors [3,7].

Previous bariatric surgery represents an important risk factor. Roux-en-Y gastric bypass predisposes patients to long-term abnormalities of calcium and vitamin D metabolism because of altered gastrointestinal anatomy and impaired nutrient absorption [6]. Vitamin D deficiency and secondary hyperparathyroidism may consequently develop, reducing physiological reserve when an additional disturbance in phosphate and vitamin D metabolism occurs. At the same time, impaired oral iron absorption makes these patients more likely to require intravenous iron therapy.

The increased susceptibility of patients with previous bariatric surgery to FCM-associated hypophosphatemia has been demonstrated clinically. Schoeb et al. prospectively evaluated patients with previous bariatric surgery receiving FCM and found a substantial incidence of new-onset hypophosphatemia. The decrease in serum phosphate was associated with increased fractional urinary phosphate excretion and increased FGF23, supporting a renal phosphate-wasting mechanism [4]. A subsequent retrospective case series of patients with Roux-en-Y gastric bypass similarly demonstrated frequent reductions in plasma phosphate following FCM administration [5].

A notable feature of the present case was the severity of the accompanying hypocalcemia. The serum calcium level of 5.3 mg/dL was associated with muscle cramps, paresthesia, and perioral numbness. FGF23-mediated alterations in phosphate and vitamin D metabolism provide a plausible link between FCM-associated phosphate wasting and disturbed calcium homeostasis. In this patient, pre-existing vitamin D deficiency and impaired calcium absorption following Roux-en-Y gastric bypass likely increased susceptibility to severe hypocalcemia. The reduced 24-hour urinary calcium excretion was also consistent with reduced calcium availability, although this finding is not specific for malabsorption. Mild hypomagnesemia may have further contributed to impaired calcium homeostasis.

The elevated PTH level was consistent with secondary hyperparathyroidism in the setting of severe hypocalcemia and vitamin D deficiency. Increased PTH may further enhance renal phosphate excretion, potentially aggravating FGF23-associated phosphate loss. The interaction among increased FGF23 activity, impaired gastrointestinal mineral absorption, and secondary hyperparathyroidism may therefore explain the severity and persistence of the electrolyte abnormalities observed in this patient.

The recurrence of symptoms following initial correction also illustrates an important clinical consideration. Intravenous replacement of calcium and phosphate can temporarily correct serum concentrations without immediately reversing the underlying mechanism responsible for ongoing phosphate loss. Consequently, patients with severe FCM-associated mineral abnormalities may require prolonged supplementation and biochemical monitoring after the initial presentation.

Recognition of patients at increased risk before FCM administration may help reduce morbidity. Reported risk factors include repeated FCM administration, low baseline phosphate levels, vitamin D deficiency, hyperparathyroidism, gastrointestinal malabsorption, and previous bariatric surgery [3,4,7]. In patients with these risk factors, assessment of serum phosphate, calcium, and vitamin D status before FCM administration should be considered. Follow-up measurement of serum phosphate is particularly relevant in patients who develop fatigue, weakness, bone pain, muscle cramps, paresthesia, or other compatible symptoms after treatment.

Alternative intravenous iron formulations may also be considered in patients who have previously experienced severe FCM-associated hypophosphatemia or who have substantial risk factors for its development. Randomized trials have demonstrated that the risk of hypophosphatemia differs considerably among intravenous iron preparations [1,2]. The choice of formulation should therefore take into account both the indication for intravenous iron replacement and the patient's underlying risk of disturbances in mineral metabolism.

This case emphasizes the importance of recognizing FCM-associated disturbances in mineral metabolism in patients with altered gastrointestinal anatomy. In individuals with previous bariatric surgery, the combination of FGF23-associated renal phosphate wasting, vitamin D deficiency, impaired calcium absorption, and secondary hyperparathyroidism may produce more severe and prolonged abnormalities than would otherwise occur.

## Conclusions

FCM is associated with clinically significant disturbances in mineral metabolism through FGF23-mediated renal phosphate wasting. Although hypophosphatemia is the characteristic biochemical abnormality, severe concurrent hypocalcemia may occur in susceptible patients. In this case, the profound hypocalcemia was likely multifactorial, reflecting the interaction between FCM-associated mineral disturbances and pre-existing vitamin D deficiency, impaired mineral absorption following Roux-en-Y gastric bypass, secondary hyperparathyroidism, and mild hypomagnesemia.

Patients with previous bariatric surgery or other conditions predisposing them to mineral deficiencies should be carefully assessed before FCM administration. Measurement of serum phosphate, calcium, and vitamin D should be considered in high-risk individuals, with follow-up testing when clinically appropriate, particularly following repeated FCM exposure or the development of compatible symptoms. Early recognition is important because the abnormalities may persist or recur despite initial electrolyte replacement and can result in substantial morbidity and repeated hospitalization.
